# The Sites of Mental Hospitals

**Published:** 1921-08-13

**Authors:** 


### THE EDITOR'S LETTER-BOX.

**Correspondence on all subjects is invited, but we cannot in any way be responsible for the opinions expressed.**

### The Sites of Mental Hospitals.

*To the Editor of* The Hospital.

Sir,—Dr. Montagu Lomax's book on the conditions of life in our lunatic asylums has received much notice and aroused not a little criticism of these institutions, of which a great deal is by no means fair. The management of the insane is a task of supreme difficulty, but to insinuate that brutality or unduly harsh control is the rule in our public asylums is certainly unjust. One important point I have not seen referred to. Constant efforts are made by those in charge to arouse the better elements in the patients' mentality by suitable work and frequent entertainments. But one factor militates against the recovery of many patients, the belief, namely, that they are forsaken by their friends. Their relations come to see them but rarely. This is due in many cases to the wicked and absurd idea that a relative whose mental condition necessitates confinement is a disgrace to his family, even as if he were a criminal. But in a large number of cases the infrequency of the visits of friends is due to the asylums being placed so far from the patients' homes that it is difficult and expensive for visitors to come. The majority of the patients in our public asylums are poor, and their friends cannot afford the time, the money, and often the fatigue involved in frequent visits. Much has been said, and too much cannot be said, in praise of the good work done at the Morningside Asylum, Edinburgh, under Sir Thomas Clouston. But Morningside Asylum is in a suburb of Edinburgh, and indeed the town has now extended far beyond it. At no time was it more than half an hour's journey from the centre of the city, and the fare was but a few pence. This made it easily possible for relations to visit the patients. Moreover, it gave the attendants a better opportunity than is often possessed for getting out of the asylum atmosphere, which tries the nerves of all, and of enjoying a more normal environment. It also enabled the authorities to invite outsiders to come frequently to help amuse the patients by music or theatricals, and to take part in the asylum dances. This proximity to the town made it possible to bring the atmosphere of the outside world into the asylum, with great benefit to the patients. Such methods are by no means confined to Morningside, but its wise and humane treatment was assisted by the fact that the asylum was within easy reach of the patients' friends.—Yours faithfully, "Scot."

### Diplomas in Radiography.

*To the Editor of* The Hospital.

